# The Tenth International Medical Congress

**Published:** 1890-02

**Authors:** 


### THE TENTH INTERNATIONAL MEDICAL CONGRESS.

In our Foreign Correspondence department will be found a communication from Dr. Miller, Berlin, regarding the present status of the Dental Section of the coming congress. The section Zahnheilkunde is placed among the medical specialties as No. 14, and precedes Hygiene, No. 15. There are eighteen sections in all. While the dental degree is not recognized in Germany, yet, by mutual consent of the general committee, it will for the time of the congress be recognized and placed on the same footing as the others. Communications in English are to be sent to Dr. Miller, No. 32 Voss Street, Berlin. It is to be hoped that there will be a liberal attendance upon the part of American dentists, and that a number of good papers will be sent in early. Quite a number have already signified their intention of attending the meeting. Let all join, as far as possible, in making our section a grand success.

